# Extracorporeal membrane oxygenation (ECMO) in COVID-19 patients: a pocket guide for radiologists

**DOI:** 10.1007/s11547-022-01473-w

**Published:** 2022-03-13

**Authors:** Michela Gabelloni, Lorenzo Faggioni, Dania Cioni, Vincenzo Mendola, Zeno Falaschi, Sara Coppola, Francesco Corradi, Alessandro Isirdi, Nicolò Brandi, Francesca Coppola, Vincenza Granata, Rita Golfieri, Roberto Grassi, Emanuele Neri

**Affiliations:** 1 Academic Radiology, Department of Translational Research, University of Pisa, Via Roma 67, 56126 Pisa, Italy; 2 Italian Society of Medical and Interventional Radiology, SIRM Foundation, Via della Signora 2, 20122 Milano, Italy; 3 Department of Surgical, Medical, Molecular Pathology and Critical Care Medicine, University of Pisa, Pisa, Italy; 4 Department of Radiology, IRCCS Azienda Ospedaliero Universitaria Di Bologna, 40138 Bologna, Italy; 5 Division of Radiology, Istituto Nazionale Tumori IRCCS Fondazione Pascale-IRCCS Di Napoli, 80131 Naples, Italy; 6 Division of Radiology, Università Degli Studi Della Campania Luigi Vanvitelli, 80127 Naples, Italy

**Keywords:** Coronavirus disease 19 (COVID-19), Severe acute respiratory syndrome coronavirus 2 (SARS-CoV-2), Acute respiratory distress syndrome (ARDS), Extracorporeal membrane oxygenation (ECMO), X-ray, Computed tomography

## Abstract

During the coronavirus disease 19 (COVID-19) pandemic, extracorporeal membrane oxygenation (ECMO) has been proposed as a possible therapy for COVID-19 patients with acute respiratory distress syndrome. This pictorial review is intended to provide radiologists with up-to-date information regarding different types of ECMO devices, correct placement of ECMO cannulae, and imaging features of potential complications and disease evolution in COVID-19 patients treated with ECMO, which is essential for a correct interpretation of diagnostic imaging, so as to guide proper patient management.

## Introduction

In January 2020, several cases of interstitial pneumonia were reported in Wuhan, China. Since mid-January 2020, the first cases of the disease were also found outside of China, and on 11 March 2020 the World Health Organization declared the coronavirus disease 19 (COVID-19) outbreak a pandemic [1]. A novel coronavirus called severe acute respiratory syndrome coronavirus 2 (SARS-CoV-2) was identified as the causative agent of the disease [2, 3].

The clinical spectrum of COVID-19 can range from no symptoms to critical illness, with an estimated incidence of acute respiratory distress syndrome (ARDS) among hospitalized COVID-19 patients hovering around 33% [4, 5]. While the reference standard for diagnosis is real-time reverse-transcription polymerase chain reaction (rt-PCR) test applied on respiratory tract specimens, chest multidetector computed tomography (CT) may help in the early detection, management, and follow-up of COVID-19 pneumonia [5–9]. Typical CT features of COVID-19 pneumonia include bilateral ground glass opacities with a predominant posterior and peripheral distribution, parenchymal consolidations with or without air bronchogram, interlobular septal thickening and crazy paving pattern, usually without pleural effusion or enlarged mediastinal lymph nodes [10–12].

In patients with COVID-19-unrelated ARDS, extracorporeal membrane oxygenation (ECMO) can be resorted to as a rescue therapy, allowing for temporary replacement of lung and/or heart function with artificial analogs in cardiac or pulmonary failure refractory to conventional clinical treatment [13]. The evidence collected so far in the literature has yielded mixed results, but several studies [14, 15] have reported a survival rate in patients with COVID-19-related ARDS treated with ECMO not lower compared to those with COVID-19-unrelated ARDS [16, 17]. Due to the use of ECMO in COVID-19 patients, there is an obvious need for radiologists to be familiar with the normal appearance of ECMO devices and the radiological findings of ECMO-related complications. While some articles have reported the main imaging findings in patients with ECMO [18–21], to the best of our knowledge no systematic reports of the imaging findings of ECMO in COVID-19 patients have been published to date.

Our purpose is to overview the imaging appearance of the catheters of ECMO devices in patients with COVID-19-related ARDS, as well as of potential complications that may arise secondary to either ECMO device placement or COVID-19 infection.

## ECMO: pathophysiological rationale and configurations

The ECMO circuit consists of a pump that propels the patient's blood to an oxygen membrane (allowing the blood to be enriched with oxygen and to eliminate carbon dioxide) and two access routes (one for drainage and one for return of the blood to the patient) [22, 23]. ECMO can be implemented using a venovenous (VV) or venoarterial (VA) configuration modality. VV-ECMO is used in patients with reversible respiratory failure and preserved cardiac function, whereas VA-ECMO is used in patients with cardiac failure, irrespective of lung function being preserved or not.

According to the Extracorporeal Life Support Organization (ELSO) recommendations, VV-ECMO may be utilized for patients with COVID-19 and severe respiratory failure with expected outcomes comparable to patients supported with VV-ECMO before the COVID-19 pandemic. On the other hand, VA-ECMO may be utilized for patients with COVID-19 and severe cardiac failure. However, the experience regarding VA-ECMO in COVID-19 patients is more limited [24].

In VA-ECMO, the drainage cannula is generally inserted into the internal jugular vein or into the femoral vein. The return cannula is preferentially placed into the femoral artery.

**Fig. 1:**
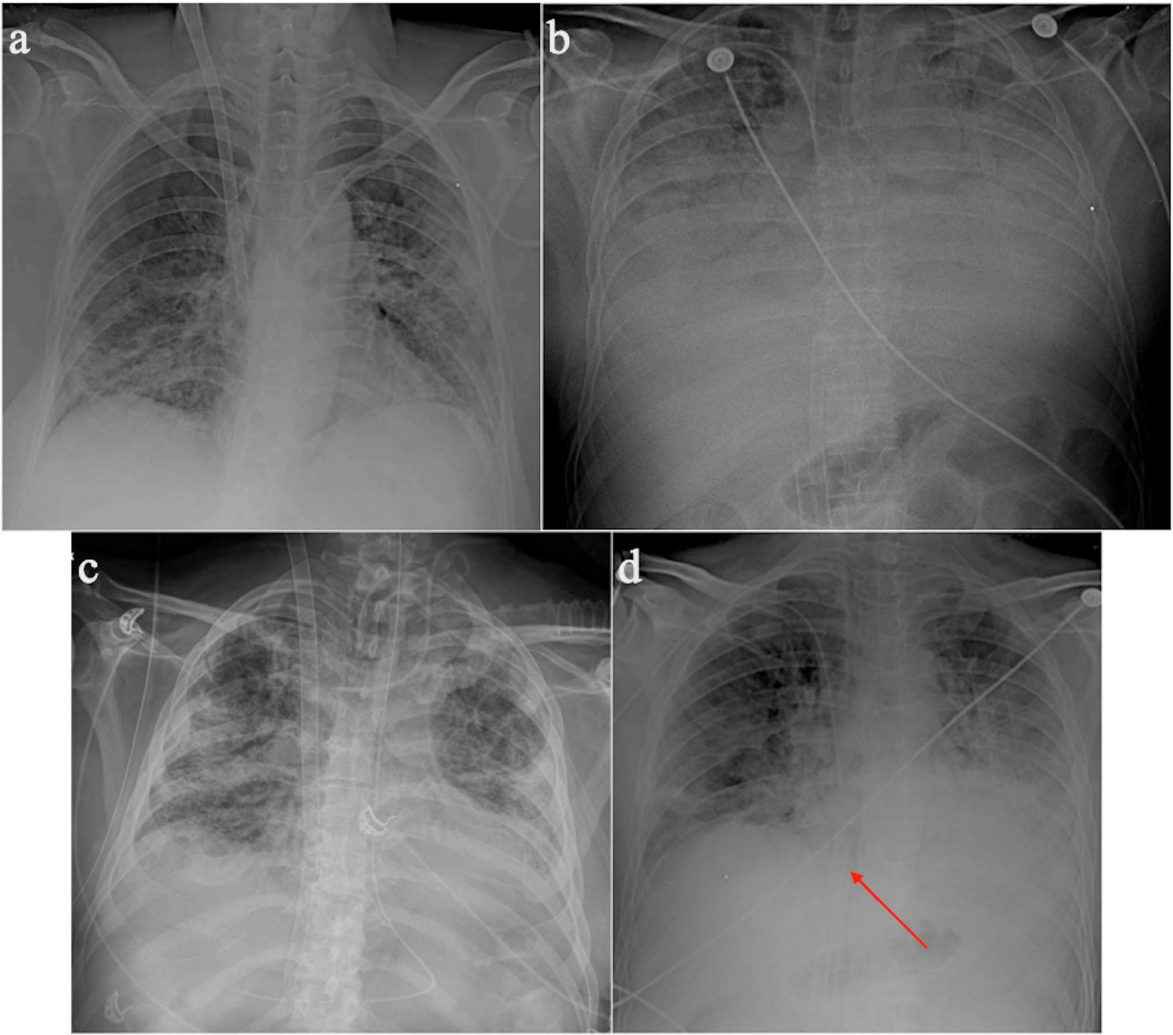
Chest radiographs (antero-posterior view) showing **a** correct placement of VV-ECMO, **b** correct placement of femorofemoral ECMO, **c** correct placement of double lumen ECMO, **d** incorrect placement of femorofemoral ECMO. The tip of the drainage cannula (arrow) is too close to the tip of the return cannula

The classic VV-ECMO configuration consists of two cannulae: a) the drainage cannula inserted into the femoral vein, with the tip at the junction between the inferior vena cava (IVC) and the right atrium, and b) the return cannula inserted into the internal jugular vein, with the tip to the level of the superior vena cava (SVC)-right atrial junction (Fig. 1a).

Another configuration is femorofemoral ECMO (also known as a double VV-ECMO cannulation), in which the drainage cannula is introduced into the femoral vein and advanced upwards until the tip reaches the distal IVC. The return cannula is inserted into the contralateral femoral vein with the tip in the right atrium (Fig. 1b).

The third configuration is the dual lumen VV-ECMO. It consists of a single cannula with a double lumen inserted from the right internal jugular vein with its tip in the IVC [25] (Fig. 1c).

According to the aforementioned ELSO guidelines, in COVID-19 patients dual lumen cannulae should be avoided if possible, as they take relatively longer time to be inserted and are associated with a higher risk of thrombotic complications and malpositioning, requiring repeat echocardiography with associated greater resource utilization and staff exposure [26].

## Positioning of ECMO cannulae: imaging findings

After the placement of ECMO cannulae, an X-ray of the chest and abdomen should be performed to evaluate their position and the distance between them, and to detect potential complications such as pneumothorax or hemothorax.

 In VV-ECMO, a distance of 15 cm between the tips of the two cannulae is recommended to avoid the phenomenon of “recirculation”, by which oxygenated blood coming to the return cannula is withdrawn through the drainage cannula without passing through the systemic circulation (Fig. 1d) [27]. A return cannula placed too inferiorly, or a drainage cannula placed too high may cause obstruction of hepatic outflow or SVC, respectively [19]. A high placement of the cannula tip in the upper SVC is associated with a higher risk of complications such as pulmonary embolism and catheter-related sepsis, whereas placement of the cannula in the RA increases the risk of cardiac tamponade. Correct positioning of the cannula is obtained when its tip is placed within the most caudal 3 cm of the SVC, but this goal is difficult to achieve. For this purpose, contrast-enhanced trans-thoracic echocardiography has been described as a useful tool for detecting tip position [28, 29].

## Imaging follow-up

According to the ELSO guidelines, ultrasonography and chest or abdomen X-rays may be performed safely at bedside as indicated, whereas multidetector CT should only be used if imaging findings may change patient management or outcome [26]. This is because CT imaging requires transferring a critically ill patient to the radiology department, thereby increasing the risk of complications (such as cannula dislodgement). However, there is no clear indication about the optimal interval at which chest X-rays should be repeated to monitor lung involvement.

**Fig. 2:**
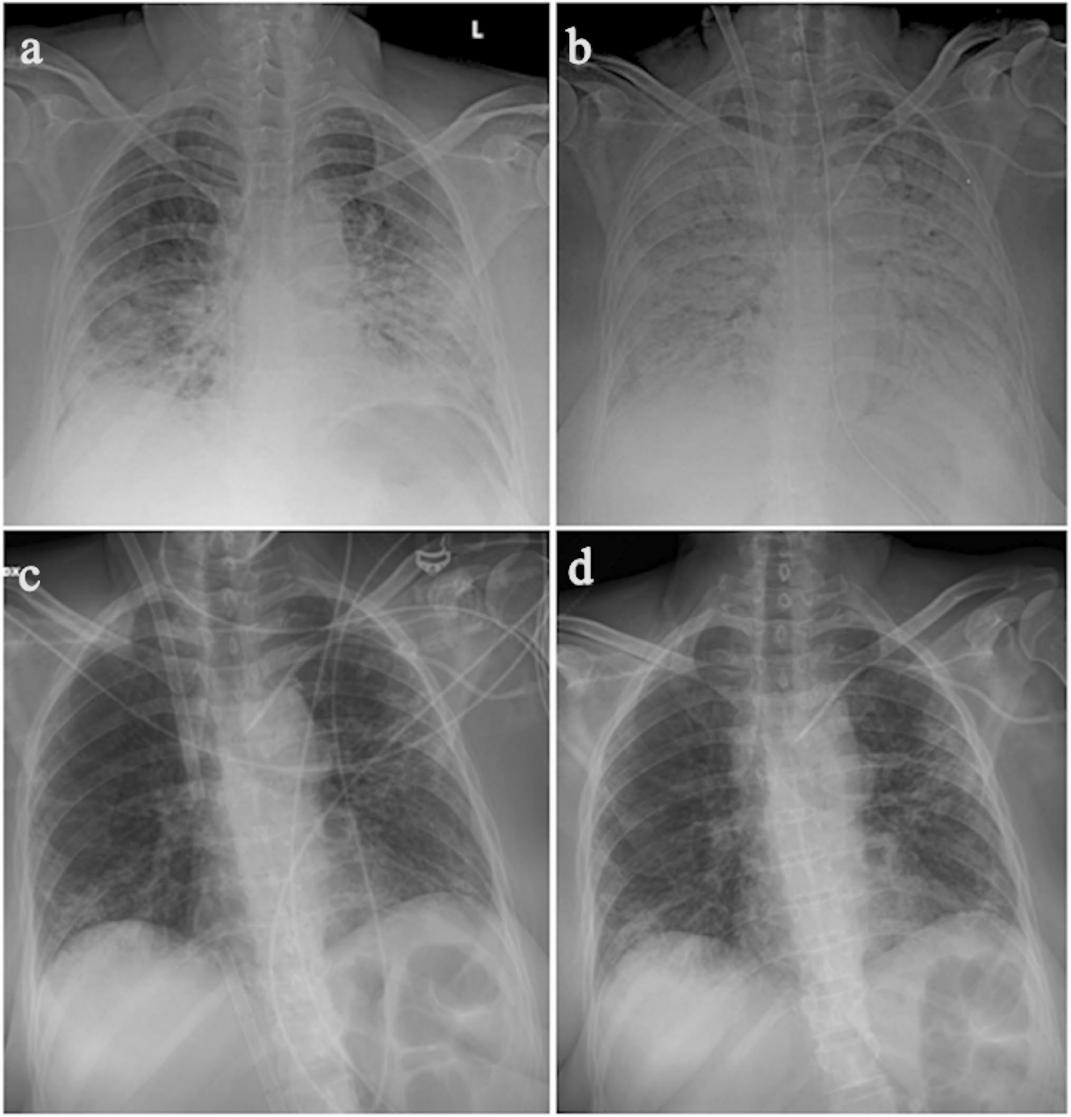
Chest radiographs **a** before and **b** after ECMO placement, showing correct placement of cannulae and complete whiteout of both lungs. **c** Chest radiographs performed at day 9 showing improvement in lung aeration and d) performed after ECMO removal, respectively

Generally, imaging of the lung parenchyma of patients with COVID-19-unrelated ARDS is of limited value in the first 7–14 days of ECMO support, due to lung opacification reflecting changes in pulmonary physiology and hemodynamics and an abrupt decrease in airway pressure. Furthermore, an improvement in lung aeration is best assessed clinically. Therefore, a chest X-ray performed immediately before and after ECMO placement, followed by chest X-ray every 2–3 days, is considered to be sufficient if the patient is stable [20] (Fig. 2).

Lung ultrasonography may represent an alternative to chest X-ray and CT [30]. Møller-Sørensen et al. found that in patients with COVID-19 treated with VV-ECMO, lung ultrasonography can distinguish patients with improving pulmonary function from those with stationary or deteriorating pulmonary function [31]. However, according to the Multinational Consensus Statement from the Fleischner Society, avoidance of non-value-added imaging is particularly important in the COVID-19 patient population to minimize exposure risk in radiology technologists and to conserve personal protection equipment [32]. In this context, it should also be taken into account that several studies have shown no difference in mortality or ventilator days for patients in the intensive care unit imaged on-demand, compared with a daily routine protocol [33–35].

## Imaging of ECMO-related complications

Complications may be related either to the patient or to mechanical factors. The latter may involve circulatory impairment, most commonly due to clots in the circuit, cannula migration/misplacement, and oxygenator failure.

In the EOLIA international clinical trial, patient-related ECMO complications were pneumothorax, acute kidney failure, cardiac arrest, neurological (e.g., hemorrhagic stroke) and infective (ventilator-associated pneumonia) complications, and bleeding [36]. In patients with COVID-19-related ARDS treated with ECMO, bleeding (42%) and thromboembolic events were quite common, with 19% having pulmonary embolism during ECMO. Nosocomial infections have also been reported frequently [37].

While chest X-ray can reveal complications such as pneumothorax or hemothorax [38], multidetector CT is the preferred modality for the assessment of thoracic complications that cannot be fully evaluated on X-rays, but also of distant complications (such as stroke or arterial/venous thrombus in large vessels). Ultrasonography and Doppler ultrasonography can be used to evaluate complications at insertion sites, including hematomas, pericannular thrombus, or deep vein thrombosis. The main complications that can be encountered in COVID-19 patients on ECMO are summarized in Table 1.

**Table 1:** Main potential complications occurring in COVID-19 patients treated with ECMO

Potential complication sites	Main clinical and radiological findings
Chest	Pulmonary embolism
	Pneumothorax
	Pneumomediastinum
	Pulmonary opacities
	Pulmonary cavitation
	Pleural effusion
Brain	Hemorrhage
	Cerebral edema
	Meningoencephalitis
	Encephalopathy
	Encephalomyelitis
Vascular	Venous thrombosis
	Bleeding
	Vessel wall dissection
	Pseudoaneurysm
	Limb ischemia
Abdomen	Pancreatitis
	Mesenteric ischemia
	Colitis
	Terminal ileitis
	Diverticulitis
	Acute renal failure

### Pneumothorax and pneumomediastinum

**Fig. 3:**
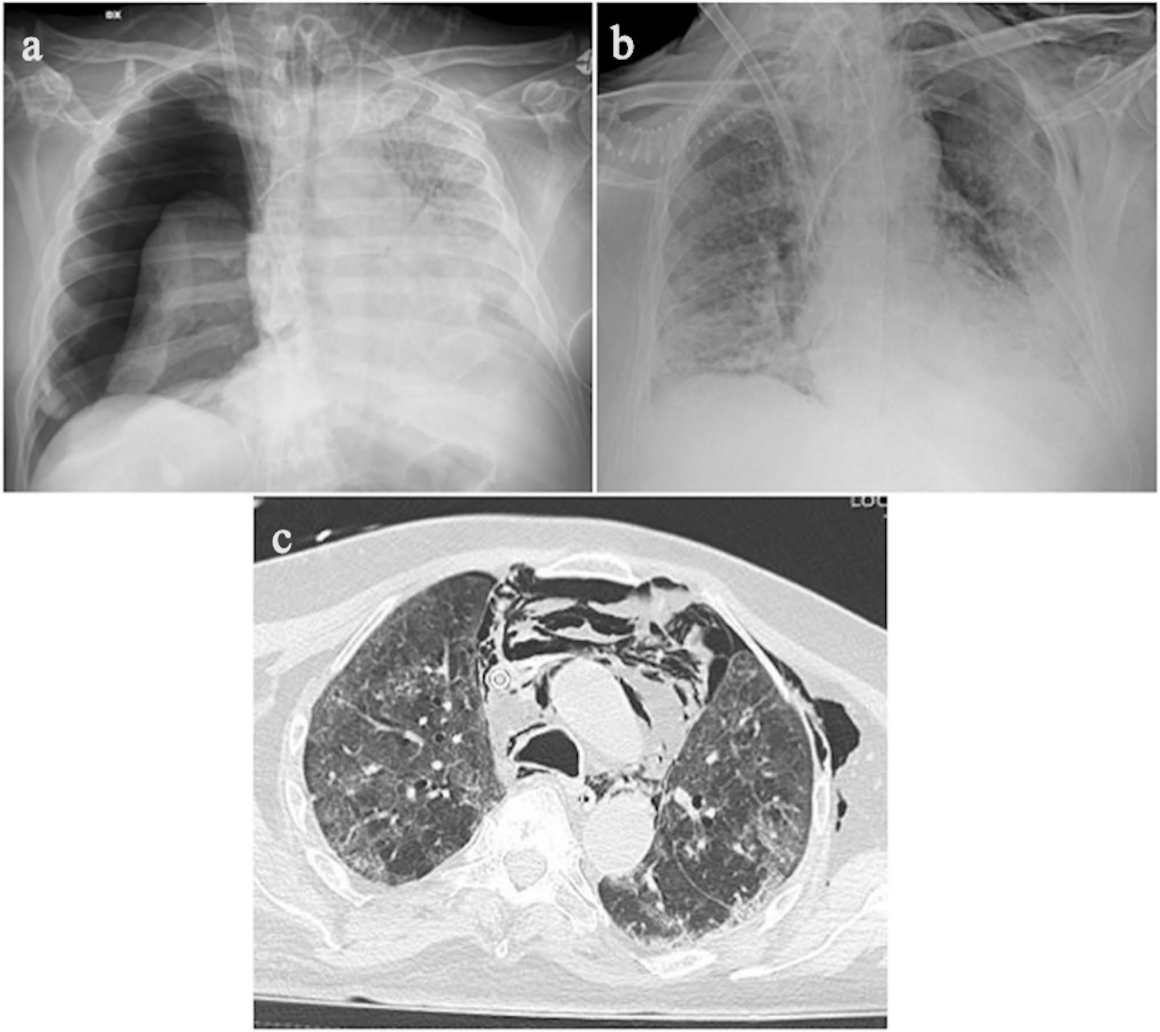
**a** Chest radiograph showing right pneumothorax. **b** Chest radiograph and **c** axial CT image showing pneumomediastinum, left pneumothorax and left subcutaneous emphysema

ECMO patients are usually also intubated and treated with high-flow ventilation, so they may be subject to barotrauma. Pneumothorax and pneumomediastinum are frequent complications in COVID-19 patients treated with high-flow ventilation (Fig. 3), as barotrauma may affect up to 15% of them [39]. Less commonly, COVID-19 patients can develop spontaneous pneumothorax and/or pneumomediastinum, as reported by Martinelli et al. in a large multicenter study. Pneumothorax can be diagnosed either incidentally or following marked respiratory deterioration characterized by hypercapnia, acidosis, and increased oxygen demand [40]. A proposed pathophysiological explanation for spontaneous pneumothorax/pneumomediastinum in COVID-19 patients involves diffuse alveolar barrier damage with consequent air leak [41], with air flowing from the pulmonary interstitium toward the pulmonary hilum and mediastinum due to the pressure gradient between the lung periphery and the mediastinal spaces [42].

### Infection

**Fig. 4:**
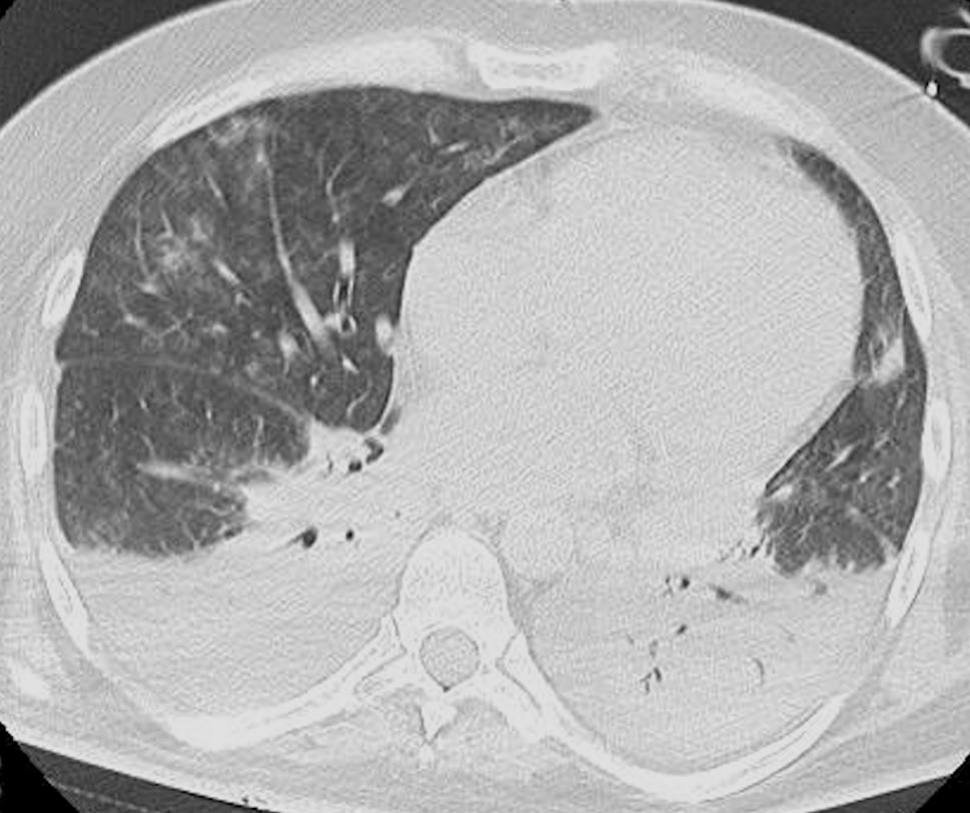
Axial CT image demonstrating bilateral lung consolidation in a COVID-19 patient with New Delhi Metallo-β-Lactamase (NDM)-producing Klebsiella pneumoniae

In COVID-19 patients, bacterial pneumonia is the most common respiratory complication (Fig. 4) [43]. Aspergillosis has been reported in 10.2% of patients admitted to the intensive care unit [44]. Bacterial infection can be diagnosed as secondary pneumonia or bacteremia during COVID-19 hospitalization and has been reported in about 6–15% of patients in whom bacterial infection had been confirmed [45, 46]. However, it is difficult to differentiate between COVID-19 pneumonia and bacterial pneumonia based on clinical features alone, and only few papers have reported the pathogen species or timing of specimen collection. The most recent guidelines from the UK National Institute for Health and Care Excellence indicate that antibiotics should only be used in COVID-19 cases if there is a clinical suspicion of bacterial infection beyond symptoms of COVID-19 pneumonia (e.g., characteristic symptoms, a neutrophil count outside the normal range, or lobar consolidation on chest imaging) [47]. In patients receiving ECMO treatment, antibiotics can be used to prevent nosocomial infection, although their efficacy remains unclear [48].

In invasively ventilated patients, ventilator-associated pneumonia is a further complication that should be considered. It is defined as a type of pneumonia that occurs 48 to 72 h after intubation in invasively ventilated patients.

### Neurological complications

Neurological complications are a broad spectrum, possibly due to viral dissemination in the central nervous system or to an inflammatory response or immune dysregulation [49]. Those associated with radiological findings include acute cerebrovascular disease, meningoencephalitis, encephalopathy, and encephalomyelitis [50, 51].

**Fig. 5:**
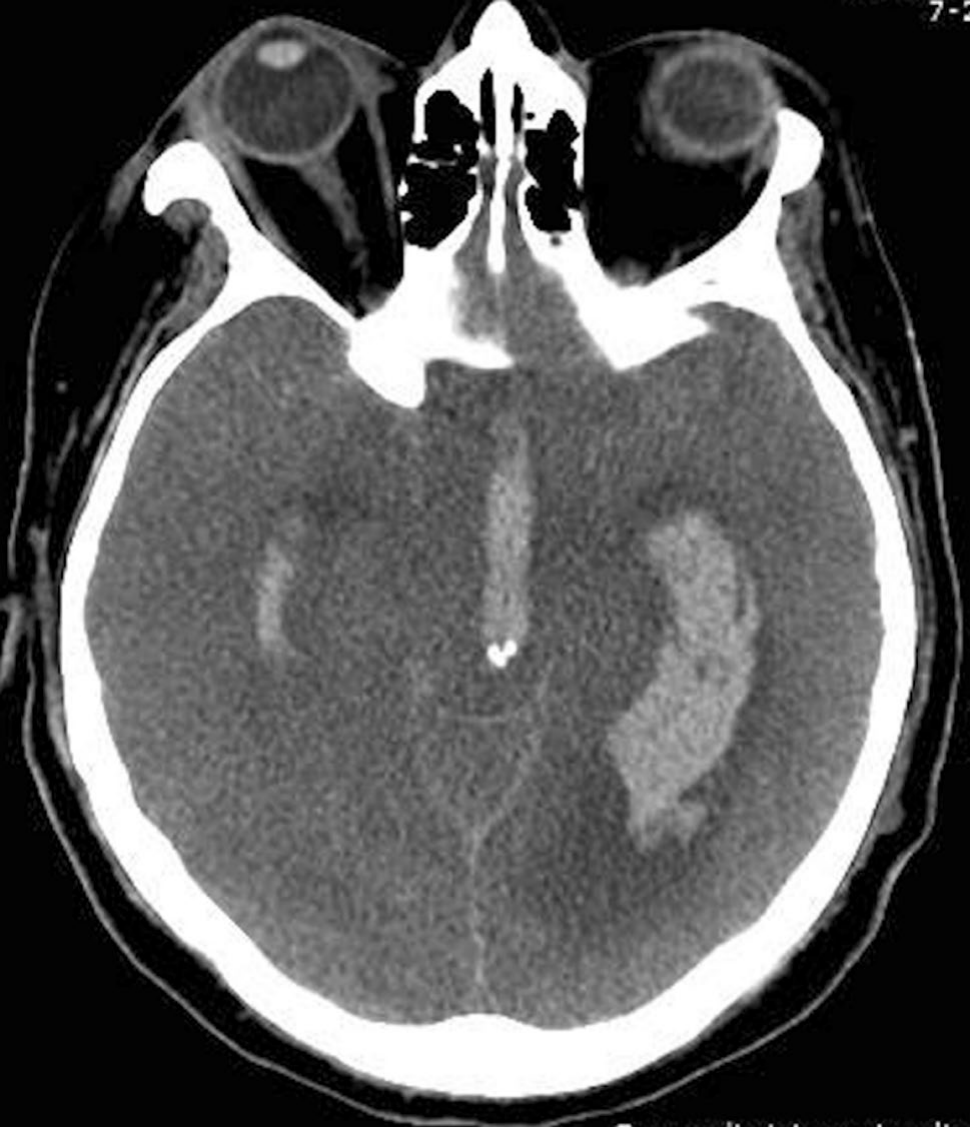
Axial CT image showing intraventricular hemorrhage in a COVID-19 patient with ECMO

During ECMO treatment, systemic anticoagulation is required to reduce circuit clotting. Consequently, one of the most common complications is extensive hemorrhage. Hemorrhagic stroke (Fig. 5) is one of the most frequent and severe neurological complications in COVID-19 patients with ECMO [52, 53]. Patients on ECMO are continuously monitored through neurological examinations (e.g., response to verbal directives or pain, brainstem reflexes, eye opening, and pupil examinations), and a head CT scan is warranted when a neurological event occurs [52].

### Hemorrhage

**Fig. 6:**
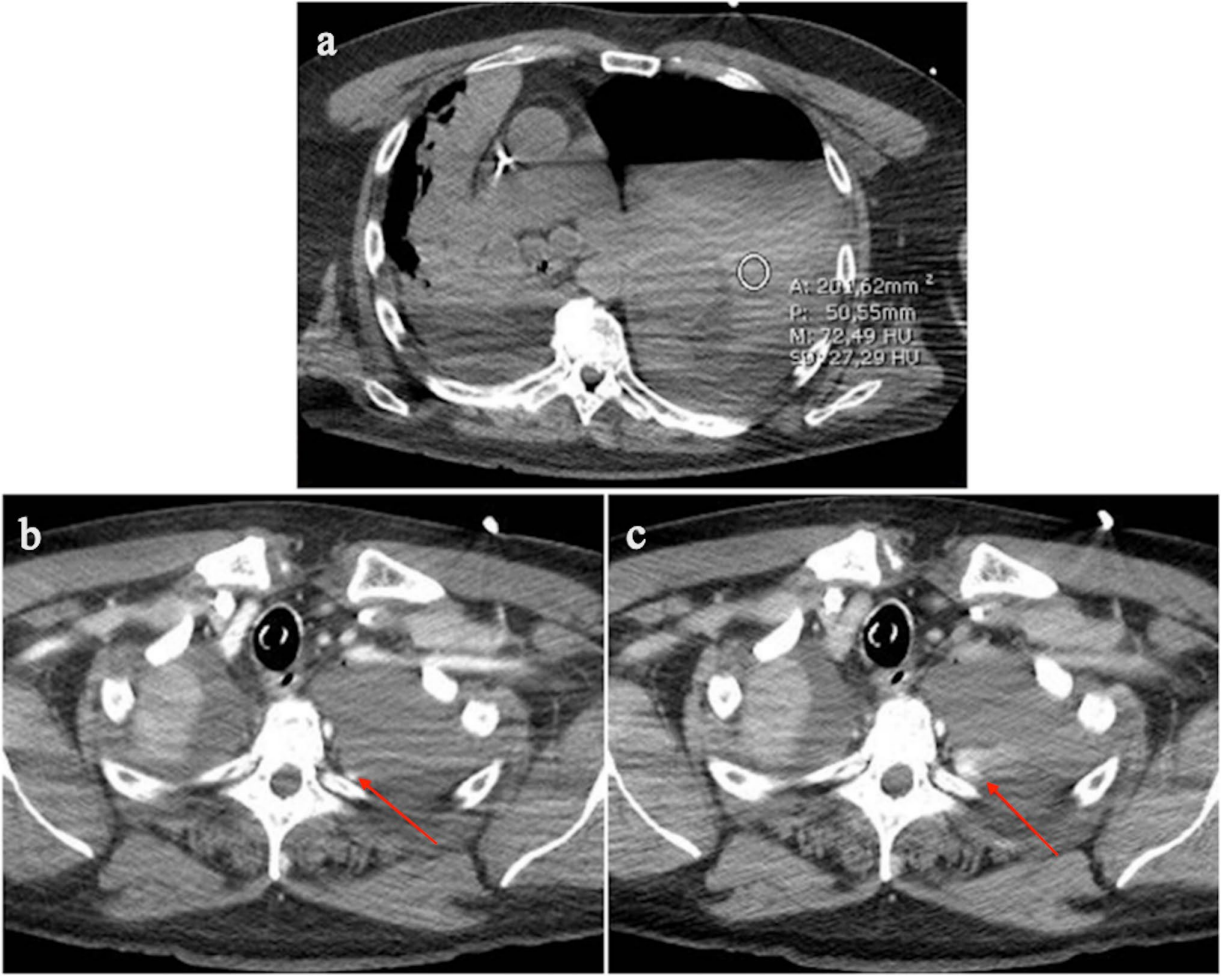
**a** Precontrast chest CT image showing hemopneumothorax in a COVID-19 patient with ECMO, rapidly decreasing hemoglobin level and hypotension. Post-contrast CT images in the **b** arterial (arrow) and **c** venous phase show active bleeding (arrow)

One of the risks related to circulatory components is the presence of clots in the circuit. Hence, anticoagulant therapy is warranted to prevent ECMO circuit from clotting and consequently to minimize the risk of thromboembolic events, such as stroke. Unfortunately, anticoagulation therapy increases the risk of hemorrhage [54]. When heparin is used as an anticoagulant, the hemostatic balance and anticoagulation are usually monitored by assessing activated clotting time (ACT), aPTT, PT, fibrinogen, anti-FXa assay, and platelet count [52, 54]. Several studies have reported intracranial cerebral hemorrhage in patients with COVID-19 [53, 55, 56]. A recent study performed on 1003 COVID-19 patients treated with ECMO has reported a 6% incidence of CNS hemorrhage [14]. The catheter insertion sites, the upper aerodigestive tract (including nose and mouth), the gastrointestinal tract, the genitourinary tract, and the thoracic and abdominal cavities are other relatively common sites of bleeding (Fig. 6). High blood pressure, low carbon dioxide blood levels, gastritis or peptic ulcer, and hematological conditions (e.g., deficiency of coagulation factors including factor XIII, thrombocytopenia, acquired von Willebrand syndrome, and platelet function defects) may cause bleeding [54].

The pathogenesis of spontaneous bleeding in patients with COVID-19 is unclear. Two possible explanations include increased serum proinflammatory cytokines and endothelial cell damage [57]. Prophylactic antithrombotic treatment is a risk factor [58].

### Vascular complications

**Fig. 7:**
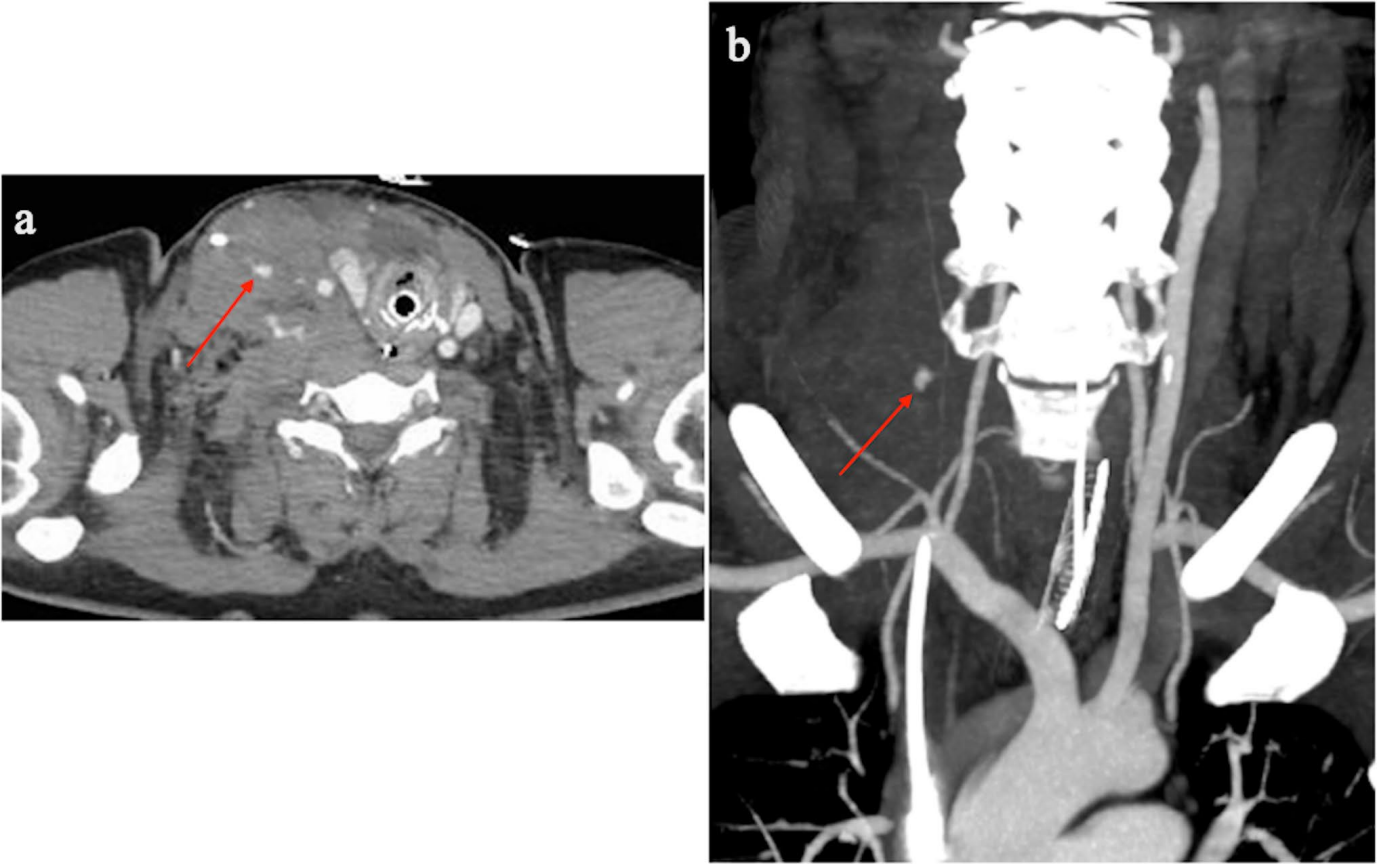
Contrast-enhanced CT showing active bleeding fed from branches of the right superior thyroid artery [**a**, axial image (arrow), **b**, maximum intensity projection (MIP) view (arrow)]

Ischemia of one or more limbs is the most common vascular complication in patients with VA-ECMO, with a reported prevalence ranging from 10 to 70%. The risk of ischemia is greater with larger cannulas (> 20Fr), in women, young patients, and in the presence of peripheral arterial disease. Possible clinical scenarios include pain, pallor, poikilothermia, motor or sensory deficit, and gangrene. Infection of the catheter insertion site, retroperitoneal bleeding, vessel wall dissection, and pseudoaneurysm formation are other common vascular complications (Fig. 7). Cellulitis, drainage around the cannula, and tissue induration can be indicators of superficial infection, whereas fever, hypotension, increased white cell count, and bacteremia are signs of systemic sepsis. Pseudoaneurysm usually presents with a painful pulsatile swelling at the access site, whereas arterial dissection is often asymptomatic (unless hemodynamically significant) [59]. In 2016, Tanaka et al. reported a 18% and 49% rate of survival to hospital discharge in patients receiving VA-ECMO with and without vascular complications, respectively [60].

### Venous thrombosis and pulmonary embolism

**Fig. 8:**
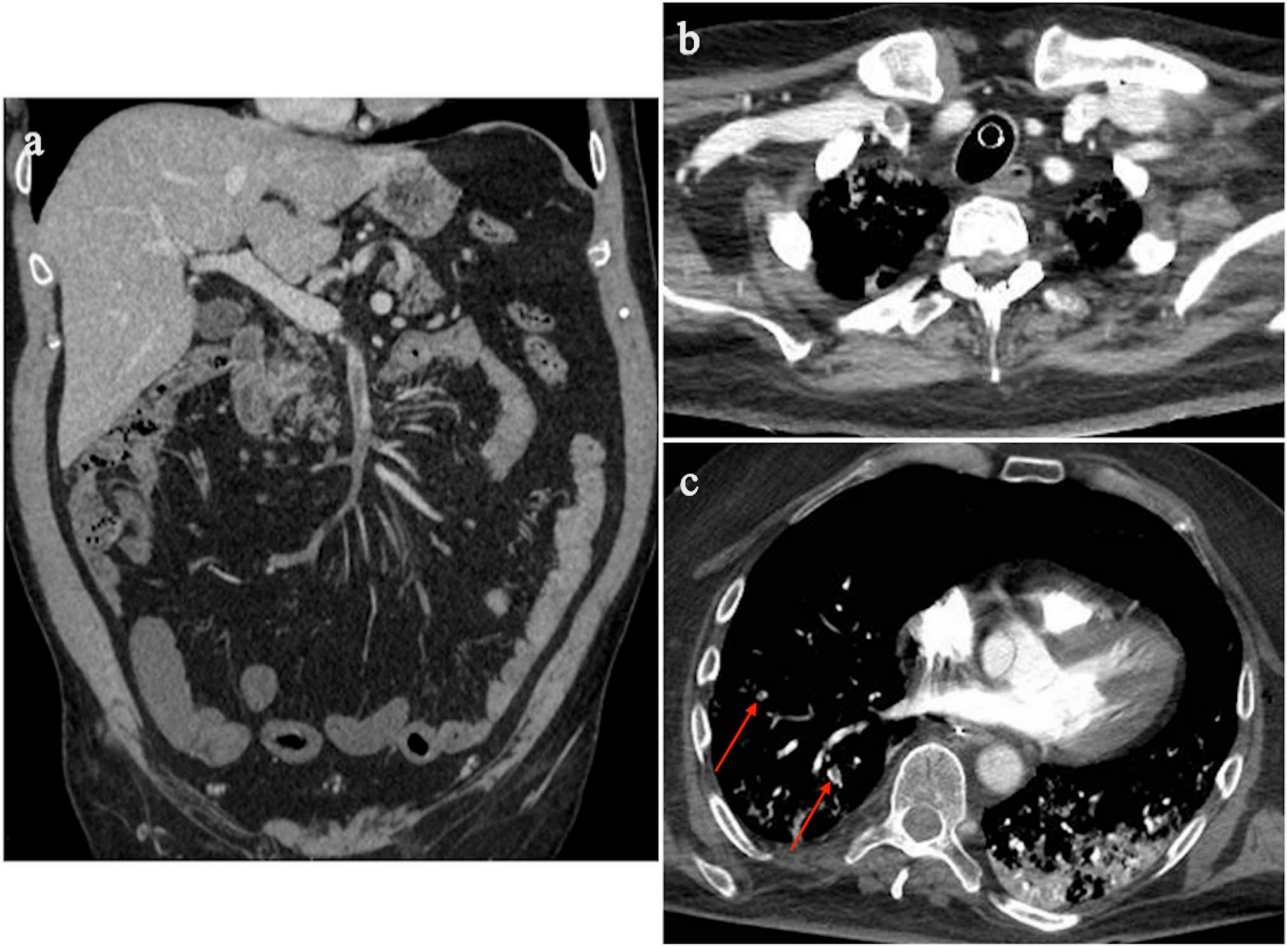
Thromboembolic complications detected incidentally on CT examinations of COVID-19 patients treated with VV-ECMO. **a** Coronal reformatted CT image of a patient with VV-ECMO and thrombosis of the superior mesenteric vein extending to several jejuno-ileal branches. **b** Axial CT image of a patient with thrombosis of the right internal jugular vein, in whom VV-ECMO had been removed two days earlier. **c** CT pulmonary angiography examination showing pulmonary embolism (arrows) at the posterior and anterior segments of the right lower lobe

COVID-19 is associated with an inflammatory state and coagulopathy, implying higher rates of venous (Fig. 8a, b) and arterial thrombosis and overall increased mortality [61–66]. A recent retrospective analysis performed on COVID-19 patients treated with ECMO has reported a 19% incidence of pulmonary embolism (Fig. 8c) [15]. On the other hand, none of the patients from the EOLIA trial developed pulmonary embolism. As a result, pulmonary embolism is probably a complication of COVID-19 disease rather than of ECMO [37]. Clinical parameters (e.g., worsening PaO2/FiO2 despite nitric oxide inhalation or after prone positioning, hemodynamic deterioration requiring fluid challenge and/or increased norepinephrine infusion rate, or dilated right ventricle without acute cor pulmonale), laboratory data (including significant or rapid elevation of D-dimer values despite anticoagulation), or both are indications for venous thromboembolism detection [62].

### Other COVID-19-related complications

**Fig. 9:**
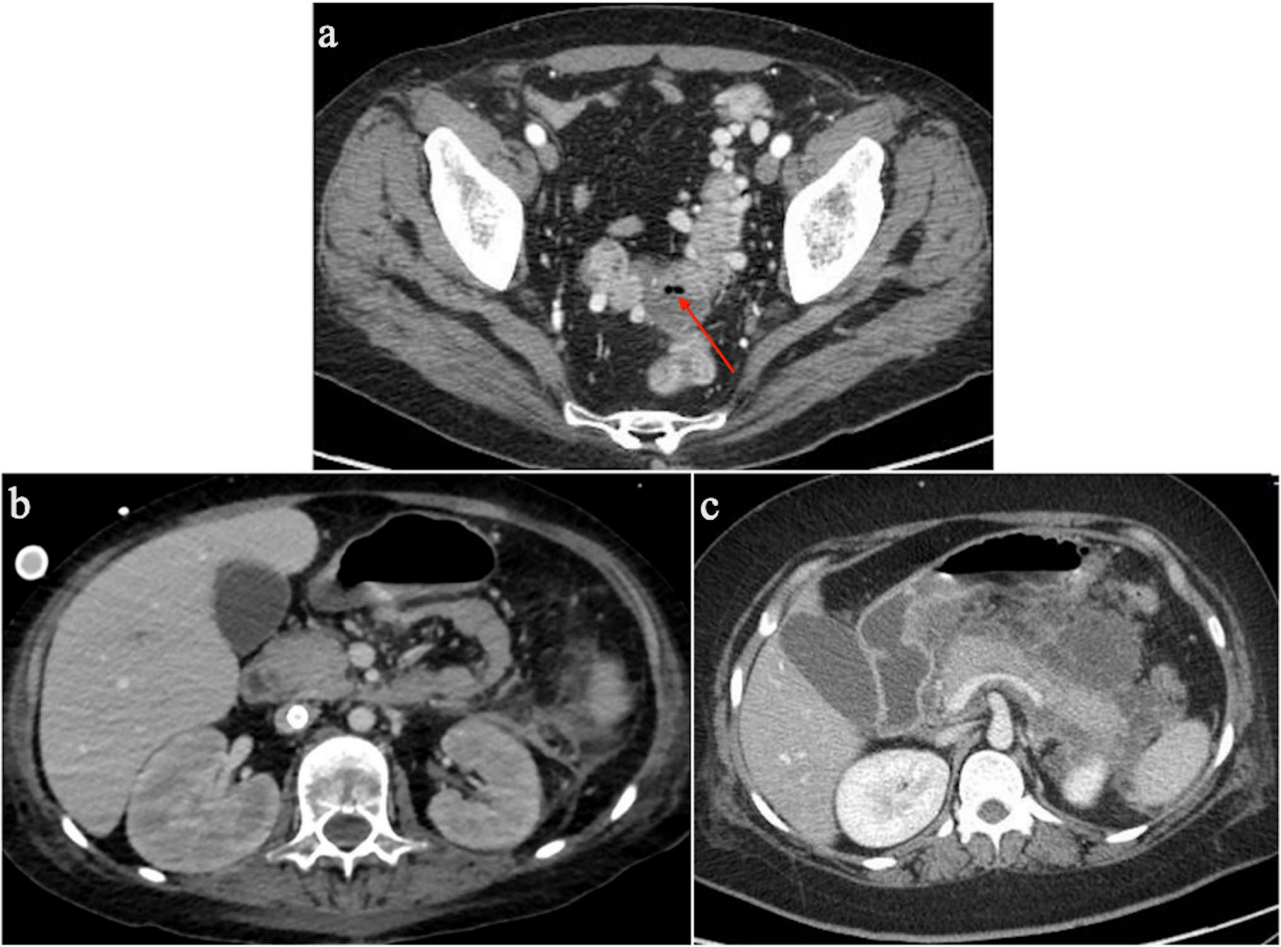
Potential abdominal complications of COVID-19 in patients treated with VV-ECMO. **a** Axial CT image shows diverticulitis complicated by a peridiverticular abscess with covered bowel perforation (arrow). **b** Axial CT image obtained in a patient with multiple organ failure and suspected abdominal bleeding. Notice initial swelling of both kidneys with loss of cortico-medullary differentiation. **c** Axial CT image obtained in a patient with peripancreatic fluid collections from acute pancreatitis

Gastrointestinal involvement is a very common extrathoracic manifestation of COVID-19, probably as a consequence of SARS-CoV-2 cytotoxicity mediated by viral binding to angiotensin converting enzyme 2 (ACE-2) receptors expressed in the gastrointestinal tract [67, 68]. Transaminitis, severe ileus, and mesenteric ischemia are the most common gastrointestinal complications [69], with diarrhea, nausea, vomiting, and abdominal pain being the most frequent gastrointestinal symptoms [70]. Colitis and terminal ileitis have also been reported as complications in patients treated in intensive care units with vasopressors and ECMO as supportive therapies [71]. COVID-19-associated diverticulitis (Fig. 9a) has also been described [72].

The presence of ACE2 receptors on kidney podocytes and proximal convoluted tubule cells could also explain the role of SARS-CoV-2 infection in the pathophysiology of acute renal failure [67]. Acute kidney injury (Fig. 9b) is one of the most common renal complications and is burdened by an increased risk of mortality ([43]).

Wang et al. [73] found a 17% incidence of pancreatic injury in patients with COVID-19 pneumonia. Pancreatitis can be diagnosed with a blood test and confirmed by clinical and radiological assessment (Fig. 9c). Unfortunately, its underlying pathophysiology is currently unknown [74].

## Conclusion

COVID-19 is a potentially life-threatening systemic disease that may require ECMO as a salvage therapy in patients who develop acute respiratory distress syndrome. Radiologists should have a comprehensive knowledge of ECMO devices and of the potential complications that may be encountered in COVID-19 patients treated with ECMO, which is essential for their proper management.
